# Improving the performance of computational ghost imaging by using a quadrant detector and digital micro-scanning

**DOI:** 10.1038/s41598-019-40798-x

**Published:** 2019-03-11

**Authors:** Ming-Jie Sun, Hao-Yu Wang, Ji-Yu Huang

**Affiliations:** Department of Opto-electronic Engineering, Beihang University, Beijing, 100191 China

## Abstract

Computational ghost imaging systems reconstruct images using a single element detector, which measures the level of correlation between the scene and a set of projected patterns. The sequential nature of these measurements means that increasing the system frame-rate reduces the signal-to-noise ratio (SNR) of the captured images. Furthermore, a higher spatial resolution requires the projection of more patterns, and so both frame-rate and SNR suffer from the increase of the spatial resolution. In this work, we combat these limitations by developing a hybrid few-pixel imaging system that combines structured illumination with a quadrant photodiode detector. To further boost the SNR of our system, we employ digital micro-scanning of the projected patterns. Experimental results show that our proposed imaging system is capable of reconstructing images 4 times faster and with ~33% higher SNR than a conventional single-element computational ghost imaging system utilizing orthogonal Hadamard pattern projection. Our work demonstrates a computational imaging system in which there is a flexible trade-off between frame-rate, SNR and spatial resolution, and this trade-off can be optimized to match the requirements of different applications.

## Introduction

Ghost imaging^1–5^, a technique closely related to single-pixel imaging^6,7^, is an alternative to conventional digital cameras based on a focal plane detector array. Ghost imaging systems use a single element detector to reconstruct images by sequentially recording the levels of correlation between the scene and a set of patterns. Digital cameras based on detector arrays perform much better in conventional visible applications, and therefore are more widely used. However, ghost imaging offers advantages in a growing range of non-conventional applications such as wide spectrum imaging^8,9^, depth mapping^10,11^ and imaging with spatially variant and reconfigurable resolution^12–14^. Yet despite these niche applications, the relatively low frame-rate and signal-to-noise ratio (SNR) of computational ghost imaging compared to imaging based on detector arrays has prevented its use from becoming more widespread.

In computational ghost imaging systems, the number of patterns required to reconstruct a fully sampled image is proportional to the total number of pixels in the reconstructed image. The sequential nature of these measurements means that increasing the system frame-rate reduces the SNR of the captured images^15^. Schemes such as differential detection^16–18^ and balanced detection^19^ have been developed to suppress system noise, and micro-scanning techniques^15,20^ have been explored to further enhance SNR. Attempts to increase the frame-rate of computational ghost imaging systems have generally focused on two strategies: (i) shortening the signal acquisition time by using fast spatial light modulators, such as digital micro-mirror devices (DMD)^8^, LED arrays^21^ or optical phase array^22^ or (ii) reducing the total number of correlation measurements required to reconstruct an image by utilizing orthogonal sampling strategies^23,24^ or compressive sensing^6,25^, i.e. under-sampling a scene and using prior knowledge of the scene such as sparsity constraints to guide the image reconstruction. Recent works have also exploited the flexibility of loading image information onto both the spatial and temporal dimensions, with the development of hybrid few-pixel computational ghost imaging systems^26,27^, for example by employing a quadrant photo-detector to increase the frame-rate by a factor of 4.

In this work, we further improve the performance of such a system by combining a hybrid few-pixel computational ghost imaging system with digital micro-scanning. Experimental results show that the proposed imaging system reconstructed images 4 times faster and with an ~33% higher SNR than a conventional single-pixel computational ghost imaging system relying on orthogonal Hadamard patterns.

## Results

**Figure 1:**
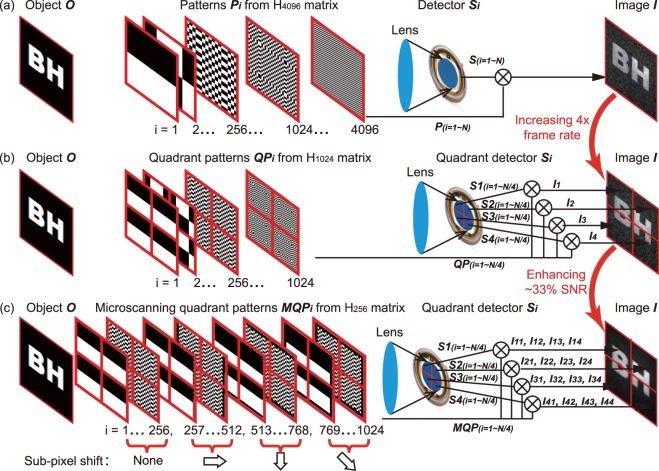
A comparison of image reconstruction schemes. (**a**) A standard computational ghost imaging system uses a single-pixel detector and takes 4096 acquisitions to yield a 64 × 64 image with a low SNR. (**b**) A few-pixel imaging system uses a quadrant detector and takes 1024 acquisitions to yield a 64 × 64 image with a low SNR. (**c**) The proposed imaging system uses a quadrant detector and takes 256 acquisitions to yield a 32 × 32 image with a high SNR, then with four sequentially obtained 32 × 32 images, a 64 × 64 image can be reconstructed.

In standard single-element computational ghost imaging (henceforth referred as CGI), as shown in Fig. 1(a), to reconstruct an image of 64 pixel × 64 pixel resolution, a set of binary patterns, each of the same resolution as the final image (i.e. 64 × 64 pixels) is generated to sample a scene. In this case the scene consists of a binary transmissive object *O*. The patterns may be either randomly generated and therefore partially correlated^3,22,28^ or form an orthonormal basis^8,9^, the latter is convenient to efficiently fully sample the scene^11–15,19–21,23,24^. One such orthonormal basis is derived from the Hadamard matrix, a square matrix with elements ±1 whose rows (or columns) are orthogonal to one another^29,30^. A set of 4096 patterns can be generated by reshaping the *i*^th^ row (or column) of a 4096 × 4096 Hadamard matrix (hereafter donated as H_4096_) into a square 64 × 64 pixel pattern *P*_*i*_. The *i*^*th*^ measurement is performed by projecting pattern *P*_*i*_ onto the scene, and measuring the intensity of the total reflected signal, *S*_*i*_, with a single element detector. *S*_*i*_ is directly proportional to the overlap integral between the pixelated object *O* and the pattern *P*_*i*_. Because these Hadmard patterns are orthogonal, an image *I* of 64 × 64 pixel resolution can be obtained after 4096 measurements as:

1 $$I=\sum _{i=1}^{4096}{S}_{i}\cdot {P}_{i}.$$

We note that as the required patterns contain negative elements, each measurement is performed by projecting binary patterns (consisting of elements 1 and 0), followed by the inverse (consisting of swapped elements 0 and 1). *S*_*i*_ is then found from the difference between these two measurements.

A hybrid few-pixel computational ghost imaging system has been proposed by replacing the single element detector with a quadrant detector^27^. By simultaneously acquiring information from each quadrant of the scene independently, this increased the frame rate of the system by a factor of 4. In the case of this few-pixel imaging system, shown as Fig. 1(b), the sampling patterns *QP*_*i*_ within each quadrant were drawn from a 1024 × 1024 element Hadamard matrix, H_1024_. With a collecting lens, four segments were imaged to the corresponding quadrants of the quadrant detector, and four correlation measurements *S*_*xi*_ (*x* = 1, 2, 3 and 4) are recorded simultaneously but independently. Therefore, four 32 × 32 pixel images *I*_*x*_ were obtained respectively using Eq. (1) and a 64 × 64 pixel image *I* of object *O* was yielded by stitching these four 32 × 32 quadrant images together. In this case, the number of sequential measurements recorded to create one 64 × 64 pixel image was 1024, i.e. 4 times lower than the number required using standard CGI. Frame-rate increases have also been demonstrated using higher numbers of detector elements in combination with structured illumination. For example, Herman *et al*.^26^ employed a 32 element multi-diode design, although crosstalk and non-uniformity among different segments must be accounted for.

In this work, we aim to enhance the system SNR at a given resolution by implementing digital micro-scanning in combination with the hybrid few-pixel imaging system as illustrated in Fig. 1(c). Digital micro-scanning uses a set of patterns with a lower resolution that are scanned across the scene. In the absence of noise, digital micro-scanning yields equivalent results to CGI. However, in the presence of noise caused by fluctuations in illumination levels and photo-detector response, the lower resolution of digitally micro-scanned patterns results in an improvement in SNR of the reconstructed image at the expense of a suppression in the contrast of high spatial frequencies^15^, i.e. the decreasing in resolving capability.

In our digital micro-scanning experiments, the sampling patterns *MQP*_*i*_ were drawn from a 256 element Hadamard matrix, H_256_. These patterns are then scanned across four locations, each with a half-Hadamard-pixel shift in x and/or y (axes parallel to the borders of the image), capturing four low resolution images which are then combined to generate a single higher resolution image^15^. In summary, considering just the measurements made by the upper left quadrant (*x* = 1) of the quadrant photodiode, we first obtained a 16 × 16 pixel image *I*_11_ by taking 256 measurements. We then obtain a second image of 16 × 16 resolution, *I*_12_, using the same set of patterns, each now shifted in x by a half-Hadamard pixel, taking another 256 measurements. Images *I*_13_ of a vertical shift and *I*_14_ of a diagonal shift are then obtained in the same manner. A 32 × 32 pixel image of one quadrant, *I*_1_, can then be reconstructed using two different methods: (i) *co-registered averaging*: by averaging the 16 × 16 pixel images *I*_*1–4*_, each co-registered in their laterally shifted locations on a 32 × 32 grid, or (ii) performing a *constrained matrix inversion*. The relative merits of these of these methods are discussed in more detail below. This process described above is simultaneously performed to reconstruct the other three 32 × 32 images *I*_2_, *I*_3_, and *I*_4_ from signals recorded by the other three photodiode quadrants, using the same procedures. Finally, a 64 × 64 pixel image *I* of object *O* is obtained by stitching together these four 32 × 32 images.

Mathematically, the 64 × 64 pixel image yielded by the *co-registered averaging* method is equivalent to the convolution of the 64 × 64 pixel image obtained by the standard (non-digitally micro-scanned) CGI system with a smoothing kernel:

2 $$k=\frac{1}{16}[\begin{array}{ccc}1 & 2 & 1\\ 2 & 4 & 2\\ 1 & 2 & 1\end{array}].$$

This convolution causes a modest reduction in contrast of the highest spatial frequencies in the image. However, as previous work has demonstrated^15^, the high resolution image reconstructed by the micro-scanned method has a higher SNR than that of the standard high resolution convolved with the smoothing kernel. It is worth mentioning that this SNR improvement will be more significant in the case of a severe noise or a low light level, that is, the micro-scanned method can deliver a recognizable image while the image obtained by a standard CGI system is too noisy to be recovered by post process algorithms.

More importantly, because the smoothing kernel *k* is known, this blur can be deconvolved recovering contrast in these high spatial frequencies. However, direct matrix inversion of the micro-scanned sampling matrix patterns is highly unstable and introduces high levels of noise into the reconstruction. Algorithms such as Weiner deconvolution^31^ could be used to address the problem – under the assumption of prior knowledge about the frequency content of the noise in the image. In this work, we use the *constrained matrix inversion* method^13^ to flexibly trade the recovery of high-resolution detail with reduced SNR. This method uses the smoothed image obtained using co-registered averaging itself as a constraint to suppress noise (see supplementary of ref.^13^ for a thorough discussion of this). The method incorporates a weighting factor *w* that weights how strongly the smoothed constraint is applied: high values of *w* result in a reconstruction that tends towards the co-registered averaging reconstruction. Low values of *w* result in a reconstruction that tends towards the noisy reconstruction obtained by direct matrix inversion. Therefore *w* can be tuned in post-processing to optimally recover high spatial frequencies while minimizing noise – the value of *w* will depend upon the levels of noise in the measurements. An additional benefit of the digitally micro-scanned approach is that it also delivers a sequence of low-resolution ‘preview’ images *during* the image acquisition, which would offer advantages for dynamic applications^13,15^.

**Figure 2:**
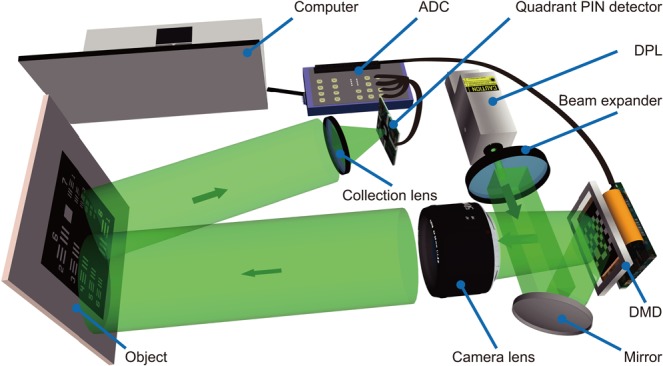
Experimental set-up. A diode pump laser (DPL) source illuminates a high-speed DMD, on which rapidly changing binary patterns are displayed. These patterns are projected by a 50 mm camera lens to illuminate a printed United States Air Force (USAF) resolution chart, which was located at a distance of ~0.5 m from the camera lens. A lens collects the reflected light, and the intensity is measured by a quadrant PIN detector. An analogue-to-digital converter (ADC), synchronized with the DMD, acquires and transfers the measured data to a computer for image reconstruction.

**Figure 3:**
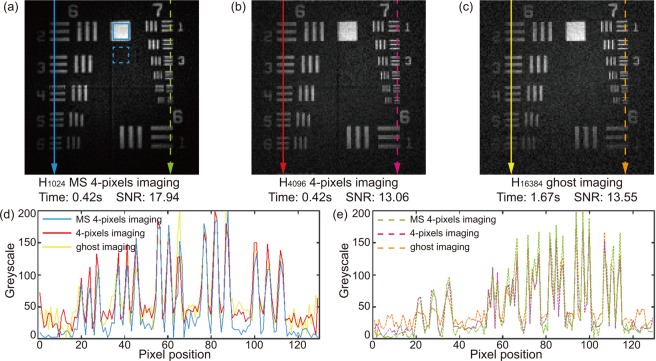
Experimental images of 128 × 128 pixel resolution obtained using (**a**) digital micro-scanning based four-pixel imaging, (**b**) Non-micro-scanning four-pixel imaging and (**c**) standard computational ghost imaging (CGI). The exposure time to capture the data for (**a**–**c**) were 0.42s, 0.42s, and 1.67s, respectively. The SNRs were calculated using data in solid and dash blue squares as features and backgrounds of the images. (**d**) Greyscale distributions highlighted by three solid lines (blue, red and yellow). (**e**) Greyscale distributions highlighted by three dash lines (green, pink and golden).

Figure 2 illustrates our CGI system utilizing digital micro-scanning and a quadrant detector. The test object was a printed United States Air Force (USAF) resolution chart, which was located ~0.5 m from the imaging system. The devices specification and their operating configurations of the experimental system are detailed in Methods. In the experiment, we sequentially displayed a set of 64 × 64 digitally micro-scanned patterns on DMD. As described above, the pattern within each quadrant was drawn from one row of the H_1024_ matrix. Each pattern was immediately followed by its inverse in order to maintain orthogonality, and to reduce fluctuation noise in ambient light by differential imaging^11^. Therefore, with the first 2048 measurements (including pattern and inverse), a 64 × 64 pixel image was reconstructed. Following the digital micro-scanning method, three more sets of 64 × 64 pixel images were reconstructed, each comprising of a further 2048 measurements, and each shifted by a half pixel displacement in x and/or y. By performing *constrained matrix inversion*, a 128 × 128 image of the test object, shown in Fig. 3(a), was reconstructed. The total number of measurements was 8192, and it took 0.42 s for data acquisition when DMD was operating at 20 kHz. The SNR of Fig. 3(a) was 17.94, which was calculated using:

3 $${\rm{SNR}}=(\langle {I}_{f}\rangle -\langle {I}_{b}\rangle )/(({\sigma }_{f}+{\sigma }_{b})/2),$$

where $\langle {I}_{f}\rangle$ and $\langle {I}_{b}\rangle$ are the average intensities of the image feature and background (here calculated from the data within the solid and dash blue square in Fig. 3a). ${\sigma }_{f}$ and ${\sigma }_{b}$, representing the noise level, are the standard deviations of the intensities in the feature and the background respectively.

For comparison, we then used the same device set-up to perform conventional (i.e. non-digitally micro-scanned) four-pixel imaging with 8192 patterns generated from the H_4096_ matrix to reconstruct a 128 × 128 image, shown in Fig. 3(b). The acquisition time was 0.42s. The SNR, calculated in the same manner, was 13.06. Finally, standard CGI was performed with 32768 reshaped Hadamard patterns from H_16384_ matrix. Four outputs of the quadrant PIN detector were summed, functioning as a single-pixel detector, to record the total light intensity of the corresponding pattern. The data acquisition took 1.67s to reconstruct a 128 × 128 image, as shown in Fig. 3(c), of which the SNR was 13.55.

**Figure 4:**
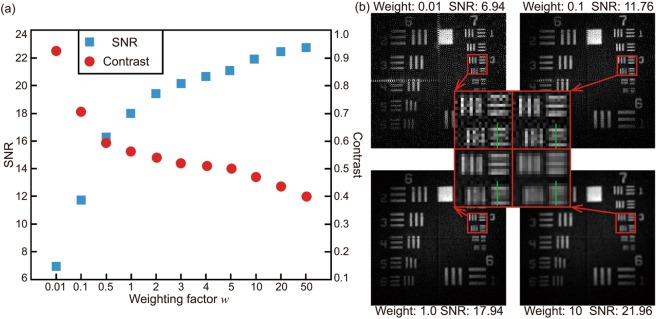
(**a**) Calculated SNRs of the images reconstructed using different weighting factors. (**b**) Examples of the reconstructed images with constraining weights at 0.01, 0.1, 1.0 and 10.

The resulting images and their calculated SNRs demonstrate that our proposed method yields an image 4 times faster than the standard single-element CGI did, and that the SNR of the image was ~33% higher than those of the images reconstructed by both 4-pixels imaging and standard CGI. Line scans along the highlighted lines in Fig. 3(a–c) are illustrated in Fig. 3(d,e), demonstrating that among the three images, the one yielded by the proposed scheme was the least noisy. However, because the proposed scheme fundamentally reconstructed image from a lower sampling resolution, the contrasts of the higher spatial frequencies are reduced in comparison with the other two methods. As described above, by tuning the weighting factor *w* of the constrained matrix inversion method (i.e. how strongly the reconstruction applies the smoothed image as a constraint), in post-processing we can trade between SNR and the contrast of the highest spatial frequencies in the reconstructed image. To illustrate this, Fig. 4(a) showed the calculated SNRs and contrasts of the images reconstructed using different weighting factors. The contrast of an image is calculated using

4 $${\rm{Contrast}}=({I}_{max}-{I}_{min})/({I}_{max}+{I}_{min}),$$

where *I*_max_ is the averaged value of three largest points along the green line in Fig. 4(b), and the *I*_min_ is the averaged value of four smallest points along the green line. The SNR increases as the weighting factor increases while the contrast decreases, which is in a good agreement with our theoretical analysis. Figure 4(b) showed four example images with zoomed high frequency details, where the contrast of the high frequency feature decreases as the weighting factor *w* and the SNR increases.

## Discussion

In this work, the SNRs are calculated from the reconstructed images, to which no noise reduction filter is applied, i.e., there is no image post process other than constrained matrix inversion. In the general sense of image processing, noise reduction filters of different sophistications are very powerful, especially with the help of the rapid developing of deep learning and other artificial intelligence algorithms. However, one problem posed by using post processing filter is that different algorithms are suitable for different type of images, while the system we proposed here increases the image SNR indiscriminately during the image acquisition stage, rather than levels all the problems for the post process algorithms to deal with.

In conclusion, we have demonstrated that by utilizing digital micro-scanning and a quadrant detector, the frame-rate and the SNR of a computational ghost imaging system can be improved simultaneously. Experimentally, the proposed system yielded images 4 times faster and with ~33% higher SNR than a standard computational ghost imaging system does, at the expense of a small reduction in resolution. Because the smoothing kernel responsible for reducing the resolution is known, it is possible to recover the highest spatial frequencies in the image, but this comes at the expense of reducing the SNR. Although here we have demonstrated the concept with a structured-illumination imaging scheme, this work is applicable to computational imaging systems based on either structured illumination or structured detection (i.e. passive modulation of the image with uniform illumination – otherwise known as single-pixel cameras). Our work demonstrates a flexible trade-off between frame-rate, SNR and spatial resolution in a computational ghost imaging system, providing the opportunity for optimization to suit the requirements of different applications.

## Methods

The experimental system (Fig. 2) used in this work is described as follow. A green laser beam emitted from a diode pump laser source (wavelength: 532 ± 0.1 nm; 200 mW) was expanded and reflected to illuminate the DMD (Texas Instruments Discovery™ 4100, 1024 × 768 pixels, with ViALUX Hi-Speed V-7000 module capable of storing all required patterns) operating at 20 kHz. The DMD displayed a preloaded sequence of binary patterns, which were projected through a camera lens (f = 50 mm; F = 1.4D) onto the test object to provide structured illumination. The reflected light was collected by a singlet lens (f = 25 mm, F/1), and the intensity measured by four segments of a quadrant PIN detector (400–1100 nm, active area: 12 mm^2^/segment). A high dynamic range analogue-to-digital converter sampling with four analog input channels at 500 kSs-1/channel, and synchronized with the DMD, acquired and transferred the intensity data to a computer to reconstruct the image. The reconstruction protocol is described in Results.

## References

[1] Pittman TB, Shih YH, Strekalov DV, Sergienko AV (1995). Optical imaging by means of two-photon quantum entanglement. Rhys. Rev. A.

[2] Bennink RS, Bentley SJ, Boyd RW (2002). Two-photon’ coincidence imaging with a classical source. Rhys. Rev. Lett..

[3] Shapiro JH (2008). Computational ghost imaging. Phys. Rev. A.

[4] Chen XH, Liu Q, Luo KH, Wu LA (2009). Lensless ghost imaging with true thermal light. Opt. Lett..

[5] Li H, Shi J, Zeng G (2013). Ghost imaging with nonuniform thermal light fields. J. Opt. Soc. Am. A.

[6] Duarte MF (2008). Single-pixel imaging via compressive sampling. IEEE Signal Proc. Mag..

[7] Bromberg Y, Katz O, Silberberg S (2009). Ghost imaging with a single detector. Phys. Rev. A.

[8] Radwell N (2014). Single-pixel infrared and visible microscope. Optica.

[9] Edgar MP (2015). Simultaneous real-time visible and infrared video with single-pixel detectors. Sci. Rep..

[10] Howland GA, Lum DJ, Ware MR, Howell JC (2013). Photon counting compressive depth mapping. Opt. Express.

[11] Sun MJ (2016). Single-pixel three-dimensional imaging with a time-based depth resolution. Nat. Commun..

[12] Aβmann M, Bayer M (2013). Compressive adaptive computational ghost imaging. Sci. Rep..

[13] Phillips DB (2017). Adaptive foveated single-pixel imaging with dynamic supersampling. Sci. Adv..

[14] Sun MJ, Zhao XY, Li LJ (2018). Imaging using hyperuniform sampling with a single-pixel camera. Opt. Lett..

[15] Sun MJ, Edgar MP, Phillips DB, Gibson GM, Padgett MJ (2016). Improving the signal-to-noise ratio of single-pixel imaging using digital microscanning. Opt. Express.

[16] Ferri F, Magatii D, Lugiato LA, Gatti A (2010). Differential ghost imaging. Phys. Rev. A.

[17] Sun M, He X, Li M, Wu L (2016). Thermal light subwavelength diffraction using positive and negative correlations. Chin. Opt. Lett..

[18] Song SC, Sun MJ, Wu LA (2016). Improving the signal-to-noise ratio of thermal ghost imaging based on positive-negative intensity correlation. Opt. Commun..

[19] Soldevila F (2016). Computational imaging with a balanced detector. Sci. Rep..

[20] Zhao Y, Chen Q, Sui X, Gao H (2017). Super resolution imaging based on dynamic single pixel camera. IEEE Photonics. J.

[21] Xu ZH, Chen W, Penulas J, Padgett MJ, Sun MJ (2018). 1000 fps computational ghost imaging using LED-based structured illumination. Opt. Express.

[22] Li LJ, Chen W, Zhao XY, Sun MJ (2019). Fast Optical Phased Array Calibration Technique for Random Phase Modulation LiDAR. IEEE Photonics. J.

[23] Zhang Z, Wang X, Zheng G, Zhong J (2017). Hadamard single-pixel imaging versus Fourier single-pixel imaging. Opt. Express.

[24] Sun MJ, Meng LT, Edgar MP, Padgett MJ, Radwell N (2017). A Russian Dolls ordering of the Hadamard basis for compressive single-pixel imaging. Sci. Rep..

[25] Baraniuk, R.G. Compressive sensing [lecture notes]. *IEEE Signal Proc*. *Mag*. **24**, 118–121 (2007).

[26] Herman MA, Tidman JM, Hewitt DE, Weston TH, Mcmackin L (2013). A higher-speed compressive sensing camera through multi-diode design. Proceedings of SPIE..

[27] Sun MJ, Chen W, Liu TF, Li LJ (2017). Image retrieval in spatial and temporal domains with a quadrant detector. IEEE Photonics. J.

[28] Padgett MJ, Boyd RW (2017). An introduction to ghost imaging: quantum and classical. Phil. Trans. R. Soc. A.

[29] Pratt WK, Kane J, Andrews HC (1969). Hadamard transform image coding. Proceedings of the IEEE.

[30] Sloane NJ, Harwit M (1976). Masks for Hadamard transform optics, and weighing designs. Appl. Opt..

[31] Wallace W, Schaefer LH, Swedlow JR (2001). A working person’s guide to deconvolution in light microscopy. Biotechniques.

